# Catechol-O-methyltransferase (COMT) influences the connectivity of the prefrontal cortex at rest

**DOI:** 10.1016/j.neuroimage.2012.11.059

**Published:** 2013-03

**Authors:** Elizabeth M. Tunbridge, Sarah M. Farrell, Paul J. Harrison, Clare E. Mackay

**Affiliations:** a Department of Psychiatry, University of Oxford, Oxford, UK; b Oxford Centre for Functional Magnetic Resonance Imaging of the Brain, University of Oxford, Oxford, UK

**Keywords:** Resting state network, Dopamine, Working memory, Prefrontal cortex, Polymorphism, fMRI

## Abstract

Catechol-O-methyltransferase (COMT) modulates dopamine in the prefrontal cortex (PFC) and influences PFC dopamine-dependent cognitive task performance. A human COMT polymorphism (Val^158^Met) alters enzyme activity and is associated with both the activation and functional connectivity of the PFC during task performance, particularly working memory. Here, we used functional magnetic resonance imaging and a data-driven, independent components analysis (ICA) approach to compare resting state functional connectivity within the executive control network (ECN) between young, male COMT Val^158^ (n = 27) and Met^158^ (n = 28) homozygotes. COMT genotype effects on grey matter were assessed using voxel-based morphometry. COMT genotype significantly modulated functional connectivity within the ECN, which included the head of the caudate, and anterior cingulate and frontal cortical regions. Val^158^ homozygotes showed greater functional connectivity between a cluster within the left ventrolateral PFC and the rest of the ECN (using a threshold of Z > 2.3 and a family-wise error cluster significance level of p < 0.05). This difference occurred in the absence of any alterations in grey matter. Our data show that COMT Val^158^Met affects the functional connectivity of the PFC at rest, complementing its prominent role in the activation and functional connectivity of this region during cognitive task performance. The results suggest that genotype-related differences in prefrontal dopaminergic tone result in neuroadaptive changes in basal functional connectivity, potentially including subtle COMT genotype-dependent differences in the relative coupling of task-positive and task-negative regions, which could in turn contribute to its effects on brain activation, connectivity, and behaviour.

## Highlights

► We studied the impact of COMT Val158Met genotype on resting state connectivity. ► We compared resting state functional connectivity in Val/Val vs. Met/Met men. ► We focussed on the predominantly prefrontal (PFC) executive control network (ECN). ► The ECN was identified using a group ICA approach. ► We found greater resting PFC functional connectivity in Val/Val vs. Met/Met men.

## Introduction

Catechol-O-methyltransferase (COMT) modulates dopaminergic transmission in the prefrontal cortex (PFC) (Tunbridge et al., 2004). The human COMT gene contains a well-studied functional polymorphism (Val^158^Met) in its sequence: the Val^158^ allele encodes a more active isoform than the Met^158^ allele (Chen et al., 2004). Consistent with prefrontal dopamine's key role in cognition (Cools and D'Esposito, 2011; Goldman-Rakic et al., 2000), the Val^158^Met polymorphism has been associated with human executive function, particularly working memory (Egan et al., 2001; Farrell et al., 2012). Pharmacological COMT inhibition is also beneficial to cognitive function, albeit in a COMT genotype-dependent manner; thus, COMT inhibition has received attention as a therapeutic target for cognitive dysfunction (Apud et al., 2007; Farrell et al., 2012; Giakoumaki et al., 2008). Furthermore, rodent studies have consistently shown that low COMT activity is beneficial in terms of cognitive performance (Lapish et al., 2009; Papaleo et al., 2008; Tunbridge et al., 2004). Notably, although it has primarily been studied with respect to executive function, COMT's impact is not limited to this domain: emerging evidence also implicates it in emotional (Drabant et al., 2006) and reward (Farrell et al., 2012; Frank et al., 2007; Tunbridge et al., 2012) processing.

The COMT Val^158^Met polymorphism is robustly linked with the PFC blood oxygen level-dependent (BOLD) response during the performance of working memory tasks (Egan et al., 2001; Mier et al., 2010): the Val^158^ allele is associated with greater PFC BOLD response for a given level of performance; a pattern of activation described as representing ‘inefficient’ PFC function (Egan et al., 2001). However, the opposite relationship (Met^158^ > Val^158^) is seen during emotional processing (Mier et al., 2010). COMT's impact on brain activation during task performance is not limited to the PFC, extending, for example, into the cingulate cortex, hippocampus, amygdala and ventral striatum (e.g. (Drabant et al., 2006; Tunbridge et al., 2012), consistent with data implicating it in neurotransmission in regions outside of the PFC (Bilder et al., 2004; Laatikainen et al., 2012)). It also modulates the functional connectivity between brain regions during task performance, although its precise impact varies as a function of the task employed and the neural circuitry involved (Bertolino et al., 2006; Drabant et al., 2006; Sambataro et al., 2009; Tan et al., 2007).

Although most studies focus on task-related brain activation, spontaneous (‘resting’) neuronal activity accounts for the main proportion of the brain's energy expenditure (Raichle and Gusnard, 2002). Patterns of spatial and temporal coherence in the spontaneous fluctuations in BOLD signal form functionally-relevant resting state networks (RSNs) (Biswal et al., 1995), the coherence of which is determined, at least in part, by genetic factors (Glahn et al., 2010). However, few individual genes contributing to RSN functional connectivity have yet been identified (Filippini et al., 2009). Given its robust impact on task-related PFC activation and connectivity, COMT is an attractive candidate for regulating RSN connectivity, particularly with respect to the executive control network (ECN), a PFC network implicated in a broad range of cognitive and emotional functions (Smith et al., 2009), thereby mirroring the domains with which COMT is linked. Here, we examined the effect of the Val^158^Met polymorphism on functional connectivity using a data-driven approach. We specifically focused on the ECN given the key role of COMT in prefrontal function, and investigated whether grey matter volume contributed to any differences in resting connectivity.

## Methods

### Participants

Healthy males aged 18 to 50 years old were recruited by advertisement. We limited our study to men given sexual dimorphisms in COMT's function (Tunbridge and Harrison, 2011). Exclusion criteria included a history of psychiatric or neurological disease, or any current use of medication. These criteria were based on reports obtained from participants and their primary care physician (general practitioner). In addition, current symptoms of depression and anxiety were assessed using the Beck Depression Inventory (BDI) and the State-Trait Anxiety Inventory (STAI). All subjects denied the use of illicit drugs. Volunteers were genotyped for the COMT Val^158^Met polymorphism using the appropriate Taqman® SNP Genotyping Assay (Applied Biosystems, Carlsbad, CA, US). Only homozygotes were selected for participation in the final study, as Val^158^ and Met^158^ homozygotes represent those with the highest and lowest COMT activity, respectively (Chen et al., 2004). The study was approved by the Oxfordshire National Health Service Research Ethics Committee B (09/H0605/69). The study population overlaps with that of our previous behavioural study (Farrell et al., 2012).

### Neuroimaging protocol

Imaging data were acquired at the University of Oxford Centre for Clinical Magnetic Resonance Imaging (OCMR) using a 1.5 T Siemens Sonata scanner (Siemens AG, Erlangen, Germany). Functional imaging consisted of 35 T2*-weighted echo-planar image (EPI) axial oblique slices that began at the cerebral vertex and encompassed the entire cerebrum and the majority of the cerebellum. A total of 160 volumes were acquired for each subject giving a total scan time of 8 min (repetition time (TR) = 3 s; echo time (TE) = 50 msec; flip angle 90°; field of view = 192 × 192; matrix size = 64 × 64). For the resting state scan, participants were asked to remain still, with their eyes closed, and not to fall asleep. A 3D T1-weighted structural scan was also acquired using a turbo fast low-angle shot sequence (TR = 12 msec, TE = 5.65 msec; voxel size = 1 mm^3^).

### Image analysis

Data were analysed using FSL (version 4.1.10) tools (www.fmrib.ox.ac.uk/fsl).

Probabilistic independent components analysis (ICA), implemented using FSL's Multivariate Exploratory Linear Decomposition into Independent Components (MELODIC; version 3.10), was used to extract the ECN. Individual pre-processing consisted of motion correction, brain extraction, spatial smoothing using a Gaussian kernel of full-width at half-maximum (FWHM) of 5 mm, and high-pass temporal filtering. Individual fMRI volumes were first registered to the individual's anatomical scan using FMRIB's Linear Image Registration Tool (FLIRT) followed by a Boundary-Based Registration (BBR) approach. Registration from anatomical to standard (MNI) space was conducted using FLIRT. The pre-processed individual functional data were temporally concatenated to produce a single 4D dataset, which was analysed by ICA using MELODIC, as described previously (Filippini et al., 2009). Automatic dimensionality estimation resulted in 17 spatio-temporal components. Components corresponding to known RSNs were identified by eye and compared to previously published maps (Smith et al., 2009), using Pearson spatial cross-correlation.

The dual regression method was employed to test the effects of COMT genotype on ECN functional connectivity (Filippini et al., 2009), with grey matter maps (obtained as detailed below) as voxel-wise covariates. The output of this dual regression was individual participant statistical maps of parameter estimates (PEs), describing the extent of every voxel's involvement in the ECN. These individual maps were incorporated into a single 4D file for each group, and genotype differences were assessed using voxel-wise non-parametric permutation-based testing (using 5000 permutations), using FSL's Randomise (version 2.1). Resulting maps were thresholded using Z > 2.3 and a family-wise error cluster significance level of p < 0.05. To further visualise the results, individual PE values were extracted from their custom ECN maps, using significant clusters as binary masks. For comparison between hemispheres, PEs from the homologous region in the opposite hemisphere were also extracted. PE values were log transformed and compared between genotype groups using analysis of variance (ANOVA) in SPSS Statistics 19 (IBM, Armonk, NY, US). Results are shown alongside the group spatial map.

Grey matter maps were assessed to investigate whether any genotype effects on functional connectivity within the ECN were confounded by gross differences in grey matter anatomy between Val^158^ and Met^158^ homozygotes. To achieve this, a whole-brain voxel-based morphometry- (VBM) style analysis was conducted using FSL-VBM (version 1.1) with default settings (Douaud et al., 2007). Briefly, structural images were brain-extracted and tissue-type segmented. The resulting grey matter partial volume images were aligned to standard space using FLIRT, followed by FMRIB's Nonlinear Image Registration Tool (FNIRT), and then averaged, modulated and smoothed with an isotropic Gaussian kernel of 3 mm. Finally, voxel-wise GLM was applied using permutation-based non-parametric testing (using 5000 permutations), correcting for multiple comparisons across space (thresholded using Z > 2.3 and a family-wise error cluster significance level of p < 0.05).

## Results

### Participants

The final sample consisted of 27 Val^158^ (aged 23.3 ± 3.8 years [mean ± S.D.]) and 28 Met^158^ (23.6 ± 7.1) male homozygotes.

### Identification of the executive control network

**Fig. 1:**
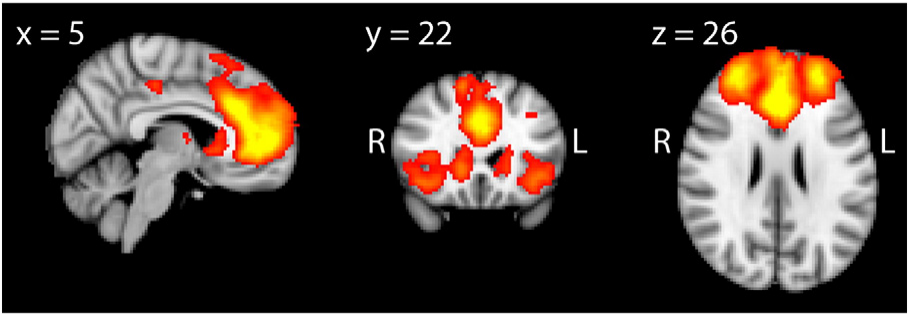
The ECN spatial map, shown thresholded at Z > 3, in the three most informative orthogonal planes and superimposed on the MNI152 standard space template image.

The group ICA successfully identified the ECN, which comprised bilateral frontal regions (encompassing the anterior cingulate cortex, anterior insula, frontal pole, and parts of the inferior and middle frontal gyri) and the head of the caudate, as well as bilateral, anti-correlated areas within the middle temporal gyrus and superior parietal lobule (Fig. 1). The ECN identified in our study was correlated (r = 0.677) with that described by Smith et al. (2009). Although not the focus of this study, the ICA identified other RSNs, which overlapped with those described by Smith et al. (2009). These included the default mode network (DMN; r = 0.749), the medial visual network (r = 0.646), the auditory network (r = 0.660), the left (r = 0.523) and right (r = 0.679) fronto-parietal networks and the sensorimotor network (r = 0.643).

### COMT genotype alters the functional connectivity of the executive control network

**Fig. 2:**
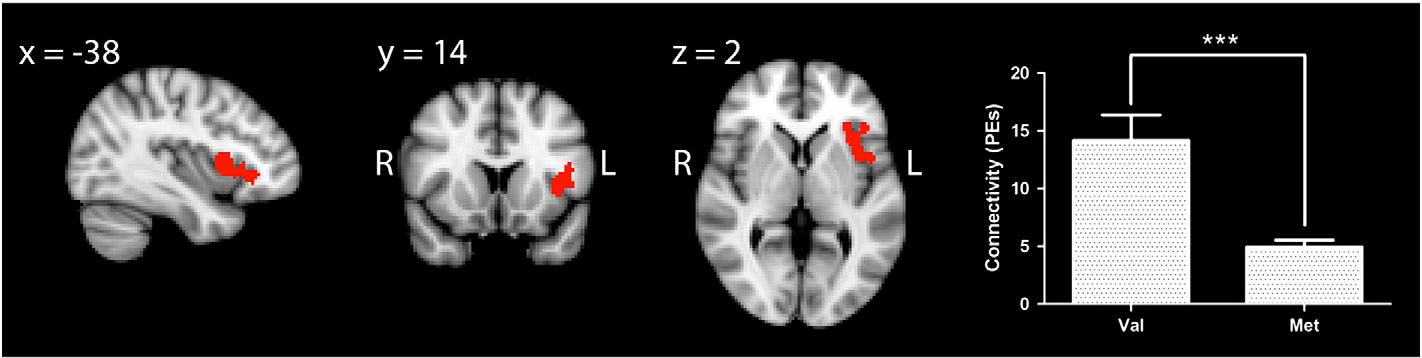
The effect of COMT Val^158^Met genotype on ECN functional connectivity. Results are shown thresholded using Z > 2.3 and a family-wise error cluster significance level of p < 0.05. The bar graph on the right of the figure shows mean PEs from within the significant cluster for Val^158^ vs. Met^158^ homozygotes. Error bars denote standard error of the mean. *** indicates p < 0.000005.

Voxel-wise comparisons of Val^158^ and Met^158^ homozygotes showed a significant effect of COMT genotype on the functional connectivity of the ECN. Specifically, Val^158^ homozygotes showed greater functional connectivity between a ventrolateral PFC (VLPFC) cluster, comprising the left insula and inferior frontal gyrus, with the rest of the ECN. The centre of gravity of this cluster is located within the left insula (MNI co-ordinates: − 38, 18, 2). Region of interest (ROI) analysis confirmed a highly significant decrease in PE values in Met^158^-, compared to Val^158^, homozygotes (F_1,53_ = 34.0; p < 0.000005; Fig. 2, which was unaffected by the inclusion or exclusion of age as a covariate). The corresponding ROI in the right hemisphere showed a decrease in PE values in Met^158^, compared to Val^158^, homozygotes, although the magnitude of this difference was smaller (Val^158^ (mean ± SEM):11.5 ± 1.8; Met^158^: 7.1 ± 0.6) than on the left, and genotype groups only differed at trend level (F_1,53_ = 3.3; p = 0.075).

We found no significant genotype differences in grey matter maps, nor did the exclusion of these maps as voxel-wide covariates substantively alter the ECN findings. Finally, exploratory analyses of the other RSNs identified by the ICA revealed no effect of COMT Val^158^Met genotype in any other network.

## Discussion

We found a significant effect of COMT Val^158^Met genotype on the functional connectivity of the ECN. Val^158^ homozygotes showed greater connectivity between the left VLPFC and the rest of the ECN, compared with Met^158^ homozygotes. Our findings complement the large body of literature demonstrating differences in brain activation and functional connectivity associated with the Val^158^Met polymorphism during task performance (Drabant et al., 2006; Egan et al., 2001; Mier et al., 2010; Sambataro et al., 2009), and show that differences in PFC function between Val^158^ and Met^158^ homozygotes exist even at rest. These changes did not appear to be due to alterations in local grey matter, as there were no between-groups differences in grey matter maps, nor were the results altered by their inclusion as a covariate.

### COMT's significance for VLPFC function

COMT's effect on brain function has predominantly been studied with respect to working memory processing. The Val^158^ allele is robustly associated with greater activation of the DLPFC for a given level of performance, compared with the Met^158^ allele, a phenomenon that has been interpreted as representing ‘inefficient’ PFC function (Egan et al., 2001; Mier et al., 2010). These findings are consistent with the critical role that dopaminergic transmission in the DLPFC plays in working memory (Goldman-Rakic et al., 2000). However, COMT's effects on cortical function are not limited to the DLPFC. Of particular relevance to the current study, there are numerous reports of COMT-related activation differences in the inferior frontal gyrus (Bertolino et al., 2006; Dennis et al., 2010; Domschke et al., 2012; Drabant et al., 2006; Ettinger et al., 2008; Green et al., 2012; Krug et al., 2009) and insula (Bishop et al., 2008; El-Hage et al., 2011; Kayser et al., 2012; Krach et al., 2010; Prata et al., 2009; Rasch et al., 2010; Schmahl et al., 2012) during the performance of a wide range of different tasks. Thus, our findings emphasise that COMT's significance extends beyond the DLPFC, and are consistent with the evidence demonstrating a role for COMT in the VLPFC.

### COMT's impact on functional connectivity at rest and during task performance

To our knowledge, only two prior studies have investigated the impact of COMT Val^158^Met genotype on connectivity at rest. Neither used a group ICA-style approach. Our results are consistent with those of Lee et al. (2011) who showed a dose-dependent relationship between the COMT Val^158^ allele and the functional connectivity of frontal regions, particularly in the left hemisphere, determined using electroencephalography. Liu and colleagues used a ROI fMRI approach to investigate COMT's effects on functional connectivity within the default mode network (DMN) (Liu et al., 2010). DMN PFC connectivity was reduced in Val^158^ homozygotes, compared with heterozygotes. Although the focus of our study was COMT's impact on the ECN, we found no impact of COMT on DMN connectivity in an exploratory analysis. However, Liu and colleagues used a longer resting state scan and a different analysis approach to that employed here and did not study Met^158^ homozygotes. Therefore, further studies of COMT's impact on DMN connectivity are warranted. It should be noted that we exclusively studied men and so it is not clear to what extent these findings will also generalise to women. However, the similarities between our findings and those of Lee et al. (2011), who studied exclusively female volunteers, is notable in this regard.

Although studies of COMT's impact on resting state connectivity are sparse, a greater number have demonstrated significant genotype effects on the functional connectivity of frontal brain regions during task performance. The regions involved and the directionality of these effects (i.e. whether it is the Val^158^ or Met^158^ allele that is associated with relatively greater connectivity) vary between these studies, presumably as the result of task differences and, accordingly, the precise neural circuitry under investigation (Bertolino et al., 2006; Sambataro et al., 2009; Tan et al., 2007). To our knowledge, only one (Sambataro et al., 2009) used a group ICA approach. Three spatial components were found to vary as a function of task demand, one of which (‘component B’) broadly overlapped with the ECN reported here. Even within this single component, COMT's effects on connectivity were complex: the frontopolar cortex showed greater connectivity associated with the Met^158^, compared with the Val^158^, allele, whilst this genotype effect was reversed in the medial superior frontal gyrus. These findings emphasise that, rather than grossly altering the overall extent of functional connectivity of the frontal cortex, COMT instead subtly modulates the precise neural circuitry used to process information. As a further example, Tan et al. (2007) demonstrated opposing effects of COMT genotype on the functional connectivity of the posterior parietal cortex (PPC) with the dorsolateral PFC (DLPFC) vs. VLPFC during performance of a working memory task. They hypothesised that the increased VLPFC-PPC connectivity seen in Val^158^ homozygotes may be a compensatory mechanism for their relatively inefficient engagement of the DLPFC-PPC circuitry, compared with Met^158^ homozygotes (Tan et al., 2007). Intriguingly, their PPC region is located within the ECN identified in our study. Therefore, our finding of greater connectivity between the VLPFC and ECN associated with the Val^158^ allele is broadly consistent with their results during working memory performance.

### Physiological relevance and links to ‘PFC efficiency’ during working memory

Successful task performance depends on engaging task-relevant (‘task-positive’) network activity, whilst suppressing that which is task-irrelevant (‘task-negative’) (Fox et al., 2005). Thus, the extent of functional connectivity between two regions during task performance can be either ‘good’ or ‘bad’, depending on whether their co-ordinated activity is beneficial or detrimental to the task in question. Although studies of the neurochemical basis of the switch between resting and task-focussed states are in their infancy, several implicate dopamine as a key neurotransmitter in co-ordinating this transition (Cole et al., 2011; Dang et al., 2012), making COMT an attractive candidate gene for modulating this process. Here, we show that Val^158^Met-associated differences in ECN connectivity are present even at rest. Interestingly, the ECN, at least as identified in the current study, comprises regions which are both task-negative (e.g. the medial PFC) and task-positive (e.g. the VLPFC) with respect to working memory (Hampson et al., 2006; Owen et al., 2005). Therefore, although caution should be exercised when linking resting-state and task-related network activity, a parsimonious explanation for our findings is that the greater functional connectivity between the VLPFC and ECN associated with the Val^158^ allele might reflect greater functional connectivity between working memory task-positive and task-negative regions. Speculatively, this super-optimal coupling at rest may detrimentally affect the brain's ability to uncouple task-positive and task-negative regions in order to perform a specific task. The inappropriate co-activation of normally task-negative PFC pathways during task performance could lead to the greater level of overall activation for a given level of performance (‘inefficiency’) observed with the Val^158^ allele.

This hypothesis is consistent with the findings of Tan et al. (2007) described above. An impaired ability to switch efficiently between resting and task-related patterns of activation has been reported previously. Notably, a dopamine transporter variant that is associated with relative deficits in cognitive function, is linked to functional connectivity between task-positive and task-negative regions, both during task performance and at rest (Gordon et al., 2012), providing direct support for the hypothesis that genetic variation in dopamine function can impact on resting vs. task-related network activity. A failure to successfully uncouple task-positive and task-negative activation has also been demonstrated in schizophrenia (Nygard et al., 2012), a disorder associated with dopamine dysfunction (Goldman-Rakic et al., 2000). Given these findings, it will be of significant interest to examine the role that COMT plays in switching between resting and task-related patterns of brain activation for different cognitive domains.

### No effect of COMT on grey matter maps

We compared grey matter (GM) maps between Val^158^ and Met^158^ homozygotes to ensure that the COMT-related alterations in ECN functional connectivity did not arise from subtle GM differences. We found no significant (or near-significant; p > 0.24) effects of COMT Val^158^Met on GM maps, consistent with some (Zinkstok et al., 2006, 2008), but not all (Ohnishi et al., 2006; Rowe et al., 2010), prior studies in healthy controls. Links between COMT and GM volume may be confounded by sexually-dimorphic effects (Zinkstok et al., 2006) and may be more prominent in populations at risk for psychosis (McIntosh et al., 2007; Ohnishi et al., 2006). Therefore, our study cannot rule out effects of COMT on GM volume in less homogeneous populations. Accordingly, we present our results corrected for GM maps (although results obtained using non-corrected data were essentially the same).

### Conclusions

In conclusion, we have demonstrated that the COMT Val^158^Met polymorphism is significantly associated with the functional connectivity of the ECN. We show that the Val^158^ allele, which is linked with PFC ‘inefficiency’ and poorer working memory performance, is associated with greater resting connectivity between the VLPFC and the ECN, compared with the Met^158^ allele. These findings complement studies showing Val^158^Met effects on functional connectivity during task performance. We speculate that aberrant connectivity between task-positive and task-negative brain regions may contribute to the relatively poorer working memory performance and PFC inefficiency associated with the Val^158^ allele.

## References

[bb0005] Apud J.A., Mattay V., Chen J., Kolachana B.S., Callicott J.H., Rasetti R., Alce G., Iudicello J.E., Akbar N., Egan M.F., Goldberg T.E., Weinberger D.R. (2007). Tolcapone improves cognition and cortical information processing in normal human subjects. Neuropsychopharmacology.

[bb0010] Bertolino A., Rubino V., Sambataro F., Blasi G., Latorre V., Fazio L., Caforio G., Petruzzella V., Kolachana B., Hariri A., Meyer-Lindenberg A., Nardini M., Weinberger D.R., Scarabino T. (2006). Prefrontal-hippocampal coupling during memory processing is modulated by COMT Val158Met genotype. Biol. Psychiatry.

[bb0015] Bilder R.M., Volavka J., Lachman H.M., Grace A.A. (2004). The catechol-O-methyltransferase polymorphism: relations to the tonic-phasic dopamine hypothesis and neuropsychiatric phenotypes. Neuropsychopharmacology.

[bb0020] Bishop S.J., Fossella J., Croucher C.J., Duncan J. (2008). COMT val158met genotype affects recruitment of neural mechanisms supporting fluid intelligence. Cereb. Cortex.

[bb0025] Biswal B., Yetkin F.Z., Haughton V.M., Hyde J.S. (1995). Functional connectivity in the motor cortex of resting human brain using echo-planar MRI. Magn. Reson. Med..

[bb0030] Chen J., Lipska B.K., Halim N., Ma Q.D., Matsumoto M., Melhem S., Kolachana B.S., Hyde T.M., Herman M.M., Apud J., Egan M.F., Kleinman J.E., Weinberger D.R. (2004). Functional analysis of genetic variation in catechol-O-methyltransferase (COMT): effects on mRNA, protein, and enzyme activity in postmortem human brain. Am. J. Hum. Genet..

[bb0035] Cole D.M., Beckmann C.F., Searle G.E., Plisson C., Tziortzi A.C., Nichols T.E., Gunn R.N., Matthews P.M., Rabiner E.A., Beaver J.D. (2011). Orbitofrontal connectivity with resting-state networks is associated with midbrain dopamine D3 receptor availability. Cereb. Cortex.

[bb0040] Cools R., D'Esposito M. (2011). Inverted-U-shaped dopamine actions on human working memory and cognitive control. Biol. Psychiatry.

[bb0045] Dang L.C., O'Neil J.P., Jagust W.J. (2012). Dopamine supports coupling of attention-related networks. J. Neurosci..

[bb0050] Dennis N.A., Need A.C., LaBar K.S., Waters-Metenier S., Cirulli E.T., Kragel J., Goldstein D.B., Cabeza R. (2010). COMT val108/158 met genotype affects neural but not cognitive processing in healthy individuals. Cereb. Cortex.

[bb0055] Domschke K., Baune B.T., Havlik L., Stuhrmann A., Suslow T., Kugel H., Zwanzger P., Grotegerd D., Sehlmeyer C., Arolt V., Dannlowski U. (2012). Catechol-O-methyltransferase gene variation: impact on amygdala response to aversive stimuli. Neuroimage.

[bb0060] Douaud G., Smith S., Jenkinson M., Behrens T., Johansen-Berg H., Vickers J., James S., Voets N., Watkins K., Matthews P.M., James A. (2007). Anatomically related grey and white matter abnormalities in adolescent-onset schizophrenia. Brain.

[bb0065] Drabant E.M., Hariri A.R., Meyer-Lindenberg A., Munoz K.E., Mattay V.S., Kolachana B.S., Egan M.F., Weinberger D.R. (2006). Catechol O-methyltransferase val158met genotype and neural mechanisms related to affective arousal and regulation. Arch. Gen. Psychiatry.

[bb0070] Egan M.F., Goldberg T.E., Kolachana B.S., Callicott J.H., Mazzanti C.M., Straub R.E., Goldman D., Weinberger D.R. (2001). Effect of COMT Val108/158 Met genotype on frontal lobe function and risk for schizophrenia. Proc. Natl. Acad. Sci. U. S. A..

[bb0075] El-Hage W., Phillips M.L., Radua J., Gohier B., Zelaya F.O., Collier D.A., Surguladze S.A. (2011). Genetic modulation of neural response during working memory in healthy individuals: interaction of glucocorticoid receptor and dopaminergic genes. Mol. Psychiatry.

[bb0080] Ettinger U., Kumari V., Collier D.A., Powell J., Luzi S., Michel T.M., Zedomi O., Williams S.C. (2008). Catechol-O-methyltransferase (COMT) val158met genotype is associated with BOLD response as a function of task characteristic. Neuropsychopharmacology.

[bb0085] Farrell S.M., Tunbridge E.M., Braeutigam S., Harrison P.J. (2012). COMT Val(158)Met genotype determines the direction of cognitive effects produced by catechol-O-methyltransferase inhibition. Biol. Psychiatry.

[bb0090] Filippini N., MacIntosh B.J., Hough M.G., Goodwin G.M., Frisoni G.B., Smith S.M., Matthews P.M., Beckmann C.F., Mackay C.E. (2009). Distinct patterns of brain activity in young carriers of the APOE-epsilon4 allele. Proc. Natl. Acad. Sci. U. S. A..

[bb0095] Fox M.D., Snyder A.Z., Vincent J.L., Corbetta M., Van Essen D.C., Raichle M.E. (2005). The human brain is intrinsically organized into dynamic, anticorrelated functional networks. Proc. Natl. Acad. Sci. U. S. A..

[bb0100] Frank M.J., Moustafa A.A., Haughey H.M., Curran T., Hutchison K.E. (2007). Genetic triple dissociation reveals multiple roles for dopamine in reinforcement learning. Proc. Natl. Acad. Sci. U. S. A..

[bb0105] Giakoumaki S.G., Roussos P., Bitsios P. (2008). Improvement of prepulse inhibition and executive function by the COMT inhibitor tolcapone depends on COMT Val158Met polymorphism. Neuropsychopharmacology.

[bb0110] Glahn D.C., Winkler A.M., Kochunov P., Almasy L., Duggirala R., Carless M.A., Curran J.C., Olvera R.L., Laird A.R., Smith S.M., Beckmann C.F., Fox P.T., Blangero J. (2010). Genetic control over the resting brain. Proc. Natl. Acad. Sci. U. S. A..

[bb0115] Goldman-Rakic P.S., Muly E.C., Williams G.V. (2000). D(1) receptors in prefrontal cells and circuits. Brain Res. Brain Res. Rev..

[bb0120] Gordon E.M., Stollstorff M., Devaney J.M., Bean S., Vaidya C.J. (2012). Effect of dopamine transporter genotype on intrinsic functional connectivity depends on cognitive state. Cereb. Cortex.

[bb0125] Green A.E., Kraemer D.J., Deyoung C.G., Fossella J.A., Gray J.R. (2012). A gene-brain-cognition pathway: prefrontal activity mediates the effect of COMT on cognitive control and IQ. Cereb. Cortex.

[bb0130] Hampson M., Driesen N.R., Skudlarski P., Gore J.C., Constable R.T. (2006). Brain connectivity related to working memory performance. J. Neurosci..

[bb0135] Kayser A.S., Allen D.C., Navarro-Cebrian A., Mitchell J.M., Fields H.L. (2012). Dopamine, corticostriatal connectivity, and intertemporal choice. J. Neurosci..

[bb0140] Krach S., Jansen A., Krug A., Markov V., Thimm M., Sheldrick A.J., Eggermann T., Zerres K., Stocker T., Shah N.J., Kircher T. (2010). COMT genotype and its role on hippocampal–prefrontal regions in declarative memory. Neuroimage.

[bb0145] Krug A., Markov V., Sheldrick A., Krach S., Jansen A., Zerres K., Eggermann T., Stocker T., Shah N.J., Kircher T. (2009). The effect of the COMT val(158)met polymorphism on neural correlates of semantic verbal fluency. Eur. Arch. Psychiatry Clin. Neurosci..

[bb0150] Laatikainen L., Sharp T., Bannerman D., Harrison P., Tunbridge E. (2012). Modulation of hippocampal dopamine metabolism and hippocampal-dependent cognitive function by catechol-O-methyltransferase inhibition. J. Psychopharmacol..

[bb0155] Lapish C.C., Ahn S., Evangelista L.M., So K., Seamans J.K., Phillips A.G. (2009). Tolcapone enhances food-evoked dopamine efflux and executive memory processes mediated by the rat prefrontal cortex. Psychopharmacology (Berl).

[bb0160] Lee T.W., Yu Y.W., Hong C.J., Tsai S.J., Wu H.C., Chen T.J. (2011). The effects of catechol-O-methyl-transferase polymorphism Val158Met on functional connectivity in healthy young females: a resting EEG study. Brain Res..

[bb0165] Liu B., Song M., Li J., Liu Y., Li K., Yu C., Jiang T. (2010). Prefrontal-related functional connectivities within the default network are modulated by COMT val158met in healthy young adults. J. Neurosci..

[bb0170] McIntosh A.M., Baig B.J., Hall J., Job D., Whalley H.C., Lymer G.K., Moorhead T.W., Owens D.G., Miller P., Porteous D., Lawrie S.M., Johnstone E.C. (2007). Relationship of catechol-O-methyltransferase variants to brain structure and function in a population at high risk of psychosis. Biol. Psychiatry.

[bb0175] Mier D., Kirsch P., Meyer-Lindenberg A. (2010). Neural substrates of pleiotropic action of genetic variation in COMT: a meta-analysis. Mol. Psychiatry.

[bb0180] Nygard M., Eichele T., Loberg E.M., Jorgensen H.A., Johnsen E., Kroken R.A., Berle J.O., Hugdahl K. (2012). Patients with schizophrenia fail to up-regulate task-positive and down-regulate task-negative brain networks: an fMRI study using an ICA analysis approach. Front. Hum. Neurosci..

[bb0185] Ohnishi T., Hashimoto R., Mori T., Nemoto K., Moriguchi Y., Iida H., Noguchi H., Nakabayashi T., Hori H., Ohmori M., Tsukue R., Anami K., Hirabayashi N., Harada S., Arima K., Saitoh O., Kunugi H. (2006). The association between the Val158Met polymorphism of the catechol-O-methyl transferase gene and morphological abnormalities of the brain in chronic schizophrenia. Brain.

[bb0190] Owen A.M., McMillan K.M., Laird A.R., Bullmore E. (2005). N-back working memory paradigm: a meta-analysis of normative functional neuroimaging studies. Hum. Brain Mapp..

[bb0195] Papaleo F., Crawley J.N., Song J., Lipska B.K., Pickel J., Weinberger D.R., Chen J. (2008). Genetic dissection of the role of catechol-O-methyltransferase in cognition and stress reactivity in mice. J. Neurosci..

[bb0200] Prata D.P., Mechelli A., Fu C.H., Picchioni M., Kane F., Kalidindi S., McDonald C., Howes O., Kravariti E., Demjaha A., Toulopoulou T., Diforti M., Murray R.M., Collier D.A., McGuire P.K. (2009). Opposite effects of catechol-O-methyltransferase Val158Met on cortical function in healthy subjects and patients with schizophrenia. Biol. Psychiatry.

[bb0205] Raichle M.E., Gusnard D.A. (2002). Appraising the brain's energy budget. Proc. Natl. Acad. Sci. U. S. A..

[bb0210] Rasch B., Spalek K., Buholzer S., Luechinger R., Boesiger P., de Quervain D.J., Papassotiropoulos A. (2010). Aversive stimuli lead to differential amygdala activation and connectivity patterns depending on catechol-O-methyltransferase Val158Met genotype. Neuroimage.

[bb0215] Rowe J.B., Hughes L., Williams-Gray C.H., Bishop S., Fallon S., Barker R.A., Owen A.M. (2010). The val158met COMT polymorphism's effect on atrophy in healthy aging and Parkinson's disease. Neurobiol. Aging.

[bb0220] Sambataro F., Reed J.D., Murty V.P., Das S., Tan H.Y., Callicott J.H., Weinberger D.R., Mattay V.S. (2009). Catechol-O-methyltransferase valine(158)methionine polymorphism modulates brain networks underlying working memory across adulthood. Biol. Psychiatry.

[bb0225] Schmahl C., Ludascher P., Greffrath W., Kraus A., Valerius G., Schulze T.G., Treutlein J., Rietschel M., Smolka M.N., Bohus M. (2012). COMT val158met polymorphism and neural pain processing. PLoS One.

[bb0230] Smith S.M., Fox P.T., Miller K.L., Glahn D.C., Fox P.M., Mackay C.E., Filippini N., Watkins K.E., Toro R., Laird A.R., Beckmann C.F. (2009). Correspondence of the brain's functional architecture during activation and rest. Proc. Natl. Acad. Sci. U. S. A..

[bb0235] Tan H.Y., Chen Q., Sust S., Buckholtz J.W., Meyers J.D., Egan M.F., Mattay V.S., Meyer-Lindenberg A., Weinberger D.R., Callicott J.H. (2007). Epistasis between catechol-O-methyltransferase and type II metabotropic glutamate receptor 3 genes on working memory brain function. Proc. Natl. Acad. Sci. U. S. A..

[bb0240] Tunbridge E.M., Harrison P.J. (2011). Importance of the COMT gene for sex differences in brain function and predisposition to psychiatric disorders. Curr. Top. Behav. Neurosci..

[bb0245] Tunbridge E.M., Bannerman D.M., Sharp T., Harrison P.J. (2004). Catechol-o-methyltransferase inhibition improves set-shifting performance and elevates stimulated dopamine release in the rat prefrontal cortex. J. Neurosci..

[bb0250] Tunbridge E.M., Huber A., Farrell S.M., Stumpenhorst K., Harrison P.J., Walton M.E. (2012). The role of catechol-o-methyltransferase in reward processing and addiction. CNS Neurol. Disord. Drug Targets.

[bb0255] Zinkstok J., Schmitz N., van Amelsvoort T., de Win M., van den Brink W., Baas F., Linszen D. (2006). The COMT val158met polymorphism and brain morphometry in healthy young adults. Neurosci. Lett..

[bb0260] Zinkstok J., Schmitz N., van Amelsvoort T., Moeton M., Baas F., Linszen D. (2008). Genetic variation in COMT and PRODH is associated with brain anatomy in patients with schizophrenia. Genes Brain Behav..

